# Can palpation-induced muscle pain pattern contribute to the differential diagnosis among temporomandibular disorders, primary headaches phenotypes and possible bruxism?

**DOI:** 10.4317/medoral.20826

**Published:** 2015-11-30

**Authors:** Yuri-Martins Costa, André-Luís Porporatti, Patrícia-dos-Santos Calderon, Paulo-César-Rodrigues Conti, Leonardo-Rigoldi Bonjardim

**Affiliations:** 1 Department of Prosthodontics, Bauru School of Dentistry, University of São Paulo, Bauru, Brazil; 2 Section of Orofacial Pain and Jaw Function, Department of Dentistry, Aarhus University, Aarhus, Denmark; 3 Department of Prosthodontics, Federal University of Rio Grande do Norte, Natal, Brazil; 4 Department of Biological Sciences, Bauru School of Dentistry, University of São Paulo, Bauru, Brazil

## Abstract

**Background:**

The evaluation of possible differences in the distribution or characteristics of palpation-induced pain in the masticatory muscles could be valuable in terms of diagnostic assessment. The aim of this study was to evaluate the impact of different combinations of anterior temporalis (AT) and masseter palpation-induced pain in the diagnostic of temporomandibular disorder (TMD), primary headaches and bruxism.

**Material and Methods:**

A total of 1200 dental records of orofacial pain adult patients were analyzed. The outcomes were dichotomously classified (presence/absence) as following: a) AT and/or masseter palpation-induced pain; b) myogenous TMD; c) temporomandibular joint (TMJ) arthralgia (arthrogenous TMD); d) migraine; e) tension-type headache (TTH); f) self-reported bruxism. Binomial logistic regression model (α = 5%) was applied to the data considering the palpation-induced muscle pain as the dependent variable.

**Results:**

Mean age (SD) were 35.7 years (13.4) for 635 included dental records (83% females). Myogenous and arthrogenous TMD, migraine, TTH and bruxism were mainly associated with, respectively, masseter palpation-induced pain (*p*<0.001 - OR=5.77, 95%CI 3.86-8.62), AT or masseter palpation-induced pain (*p*<0.001 - OR=2.39, 95%CI 1.57-3.63), bilateral AT palpation-induced pain (*p*<0.001 - OR=2.67, 95%CI 1.64-4.32), masseter and AT palpation-induced pain (*p*=0.009 - OR=1.62, 95%CI 1.12-2.33) and bilateral masseter palpation-induced pain (*p*=0.01 - OR=1.74, 95%CI 1.13-2.69).

**Conclusions:**

Palpation-induced pain in the masticatory muscles may play a role in the differential diagnosis among painful TMD, primary headaches and bruxism.

**Key words:**Diagnosis, temporomandibular joint disorders, migraine, tension-type headache, bruxism.

## Introduction

The pain assessment is a challenge task, considering that pain is a subjective experience and it is determined by a complex system of neuronal connections and it is influenced by biological, emotional and behavioral factors (1). In this scenario, the palpation-induced pain is an easy and informative psycho physical technique for the clinical assessment of deep pain (2). In particular, the diagnostic classification of painful musculoskeletal conditions, e.g., temporomandibular disorder (TMD), defined as a collection of clinical problems that involve the masticatory muscles, the temporomandibular joint (TMJ) and associated structures, are mostly based on the information of pain upon palpation of the muscles and joint (1). Furthermore, palpation-induced pain could be considered relevant for the assessment of primary headaches disorders and, in fact, it is useful to differentiate subtypes of tension-type headache (TTH), i.e., with or without pericranial tenderness (3). Finally, bruxism, a non-painful disorder, which could be defined as a sleep or wakefulness repetitive jaw-muscle activity characterized by clenching or grinding of the teeth and/or by bracing or thrusting of the mandible (4), is often associated with muscle pain and fatigue and it is considered a risk factor for TMD (5).

Researches focusing the muscle pain upon palpation or more standardized and reliable techniques, e.g., pressure pain threshold (PPT), have been elucidating some underlying mechanisms of myofascial pain and primary headaches, i.e., peripheral and central sensitization (6-8). Also, experimental tooth clenching models have unraveled the relationship between repetitive muscle activity and pain (9). However, in the clinical settings there is a lack of evidence regarding the pattern of palpation-induced muscle pain in the aforementioned disorders. Furthermore, considering the high prevalence of primary headaches, TMD, and bruxism (10-12) and their presumed triple comorbidity (13), the evaluation of possible differences in the distribution or characteristics of palpation-induced pain in the masticatory muscles could be valuable in terms of diagnostic assessment. 

Based on that, the objective of this study was to evaluate the impact of different combinations of anterior temporalis (AT) and masseter palpation-induced pain in the diagnostic of temporomandibular disorders, primary headaches phenotypes and possible bruxism.

## Material and Methods

- Design and Sample

This was a cross-sectional study with a retrospective design conducted in Brazil and performed in accordance with the ethical standards of the Declaration of Helsinki and its later amendments and approved by Ethic Committee of Human Research of Bauru School of Dentistry, University of Sao Paulo.

The sample size comprised all the population (1200 dental records) of patients who lived in the metropolitan region of Bauru and were referred or sought care for orofacial pain at the Orofacial Pain Clinic of Bauru School of Dentistry, a tertiary care center, among 1996 and 2009. Qualified professionals in training and under the supervision of two experienced professors and TMD specialists examined all the patients. The data collection occurred between 2010 and 2011. All subjects who took part in the study gave their informed consent.

All records comprised details of the clinical examination which consisted of comprehensive medical history, information about chief pain complaint, headache, intensity, frequency and quality of pain/headache, history of trauma, parafunctional habits, i.e., awake or sleep bruxism (self-report), medications intake and presence of systematic or degenerative diseases. The physical examination comprised the measurements of mandibular movements (open, lateral and protrusive) and assessment of TMJ noises and manual palpation of temporomandibular joint (TMJ), masticatory muscles and also sternocleidomastoid and trapezius.

According to the baseline clinical exam, the diagnostic algorithm of myogenous TMD (myalgia and myofascial pain) and TMJ arthralgia (arthrogenous TMD) were made according the guidelines of the American Academy of Orofacial Pain (14) whilst the diagnostic of TTH or migraine were made according the International Classification of Headache Disorders (ICHD 2) (15). Also, the diagnostic of possible bruxism was defined according the proposals for a grading diagnostic system recently published by a group of experts, that suggest the term “possible” when only anamnestic approach is accomplished (4).

The inclusion criteria were: a) adults aged 18 years or more; b) a clearly description of the assessment of AT and masseter palpation-induced pain; c) the address of parafunctional habits (sleep or awake clenching/grinding) by self-report.

The exclusion criteria were: a) presence of neurological or neuropathic diseases; b) fibromyalgia; c) systemic arthritis; d) other primary headaches than migraine or tension-type headache; e) history of intracranial disorders, vascular disorders and other major causes of headache than temporomandibular joint disorders, listed in the ICHD 2 (15), f) lack of information which avoided the diagnostic of temporomandibular disorders (TMDs), migraine and TTH. Therefore, we dealt with the missing data by excluding incomplete records.

- Variables

Based on the clinical examination and eligibility criteria, the variables of this report were retrospectively identified and dichotomously classified as following: a) presence/absence of AT and/or masseter pain to palpation; b) presence/absence of myogenous TMD; c) presence/absence of TMJ arthralgia; d) presence/absence of migraine; e) presence/absence of TTH; f) presence/absence of possible bruxism.

- Statistics

The description of age and the distribution of gender and diagnostic of myogenous TMD, TMJ arthralgia, migraine, TTH and possible bruxism were made for each group.

The inferential analysis was made through binomial logistic regression model stepwise backward considering the following dependent variables: a) AT palpation-induced pain; b) unilateral AT palpation-induced pain; c) bilateral AT palpation-induced pain; d) masseter palpation-induced pain; e) unilateral masseter palpation-induced pain; f) bilateral masseter palpation-induced pain; g) AT or masseter palpation-induced pain; h) AT and masseter palpation-induced pain; i) only AT palpation-induced pain; j) only masseter palpation-induced pain. The independent variables in all analyses were the diagnostic of (1) myogenous TMD, (2) TMJ arthralgia, (3) migraine, (4) TTH and (5) possible bruxism. We considered a 5% of significance level and 95% of Confidence Interval (CI). The Statistical Package for the Social Sciences (SPSS) v.18.0 (IBM Corp., USA) was used to perform the tests.

## Results

**Figure 1:**
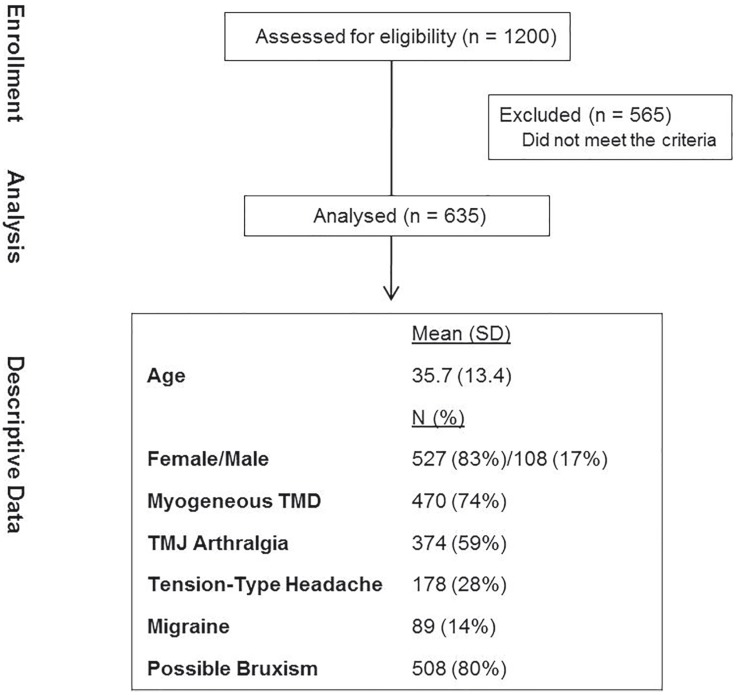
Flowdiagram and descriptive data of the study.

We analyzed 1200 dental records of which 635 fulfilled the criteria and were selected. The main reason for exclusion was the lack of information, which prevented the diagnostic of the target conditions. Demographic characteristics and diagnostic categories were: 83% were women, the mean age (SD) was 35.7 (13.4) years and 74% had myogenous TMD, 59% TMJ arthralgia, 28% TTH, 14% migraine and 80% possible bruxism (Fig. 1).

 Table 1- Table 4 show the results of the logistic regression analysis. After 1 step, only migraine did not make a significant contribution considering AT palpation-induced pain (*p*>0.05), whereas for masseter palpation-induced pain, only myogenous TMD (*p*<0.001 - OR=5.77, 95% CI=3.86-8.62) and TMJ arthralgia (*p*=0.002 - OR=1.79, 95% CI=1.23-2.59) were significantly associated after 3 steps ( Table 1).

**Table 1:**
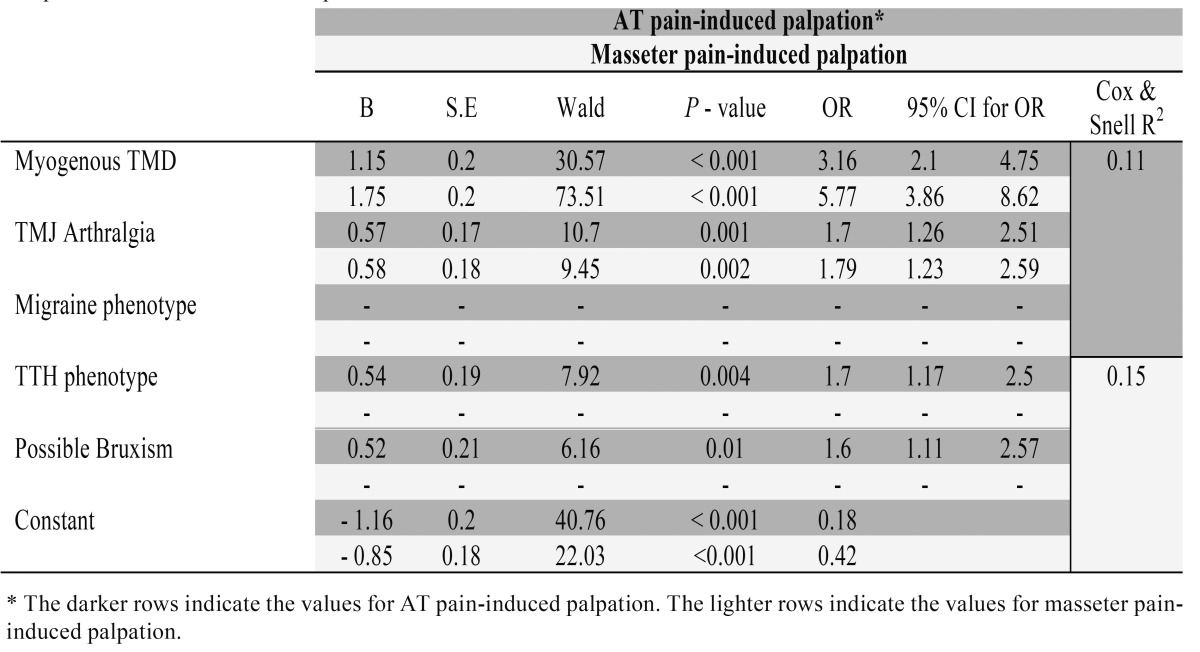
Logistic regression analysis. Anterior temporalis (AT) and masseter pain-induced palpation are the dependent variables and myogenous TMD temporomandibular joint (TMJ) arthralgia, migraine, tension-type headache (TTH) phenotypes and possible bruxism are the independent variables.

In relation to unilateral AT palpation-induced pain as the dependent variable, migraine (*p*=0.02 - OR=0.42, 95% CI=0.2-0.87) and TMJ arthralgia (*p*=0.01 - OR=1.69, 95% CI=1.11-2.59) made a significant contribution to the model after 3 steps, whereas for unilateral masseter palpation-induced pain, only myogenous TMD (*p*<0.001 -OR=3.12, 95% CI=1.89-5.14) was significantly associated after 3 steps ( Table 2).

**Table 2:**
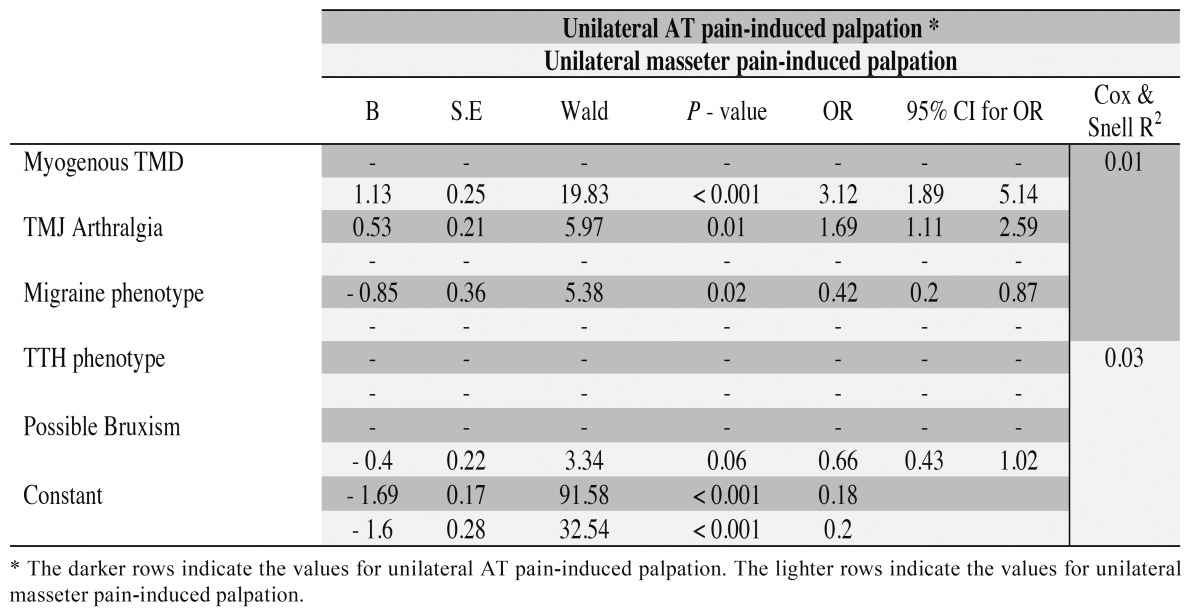
Logistic regression analysis. Unilateral anterior temporalis (AT) and masseter pain-induced palpation are the dependent variables and myogenous TMD, temporomandibular joint (TMJ) arthralgia, migraine, tension-type headache (TTH) phenotypes and possible bruxism are the independent variables.

Myogenous TMD (*p*<0.001 - OR=3.98, 95% CI=2.44-6.49), migraine (*p*<0.001 - OR=1.75, 95% CI=1.23-2.49) and TTH (*p*=0.01 - OR=1.58, 95% CI=1.09-2.3) were significantly associated with bilateral AT palpation-induced pain after 2 steps, whereas for bilateral masseter palpation-induced pain, myogenous TMD (*p*<0.001 - OR=2.43, 95% CI= 1.58-3.74), TMJ arthralgia (*p*=0.001 - OR=1.75, 95% CI=1.23-2.49) and possible bruxism (*p*=0.01 - OR=1.74, 95% CI= 1.13-2.69) made a significant contribution to the model after 1 step ( Table 3).

**Table 3:**
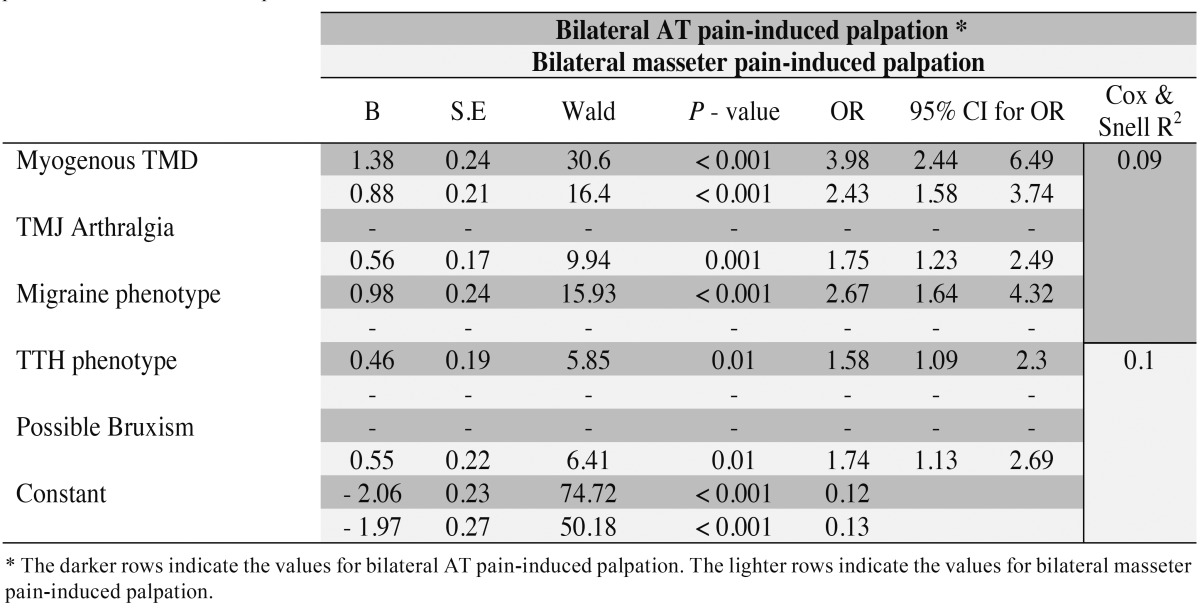
Logistic regression analysis. Bilateral anterior temporalis (AT) and bilateral masseter pain-induced palpation are the dependent variables and myogenous TMD, temporomandibular joint (TMJ) arthralgia, migraine, tension-type headache (TTH) phenotypes and possible bruxism are the independent variables.

Only migraine did not make a significant contribution considering palpation-induced muscle pain (AT and/or masseter) after 1 step. Also, TTH and possible bruxism did not make a significant contribution to the model considering AT or masseter palpation-induced pain ( Table 4). Finally, in relation to only AT palpation-induced pain, there was no significant association after 5 steps, whereas for only masseter palpation-induced pain, there was a significant association with myogenous TMD (*p*=0.001 - OR=2.28, 95% CI=1.39-3.74) after 4 steps.

**Table 4:**
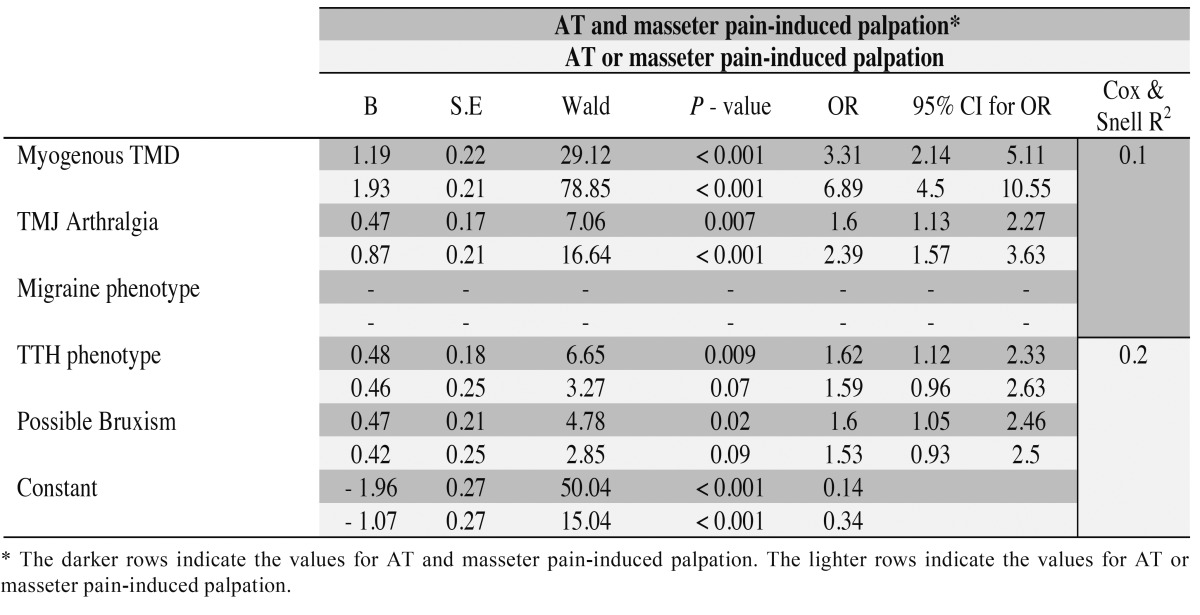
Logistic regression analysis. Anterior temporalis (AT) and/or masseter pain-induced palpation are the dependent variables and myogenous TMD, temporomandibular joint (TMJ) arthralgia, migraine, tension-type headache (TTH) phenotypes and possible bruxism are the independent variables.

## Discussion

The main findings of this retrospective study were: a) the greater the number of palpation-induced pain sites, the greater the amount of associated diagnostic categories; b) myogenous TMD was mainly associated with masseter palpation-induced pain; c) TTH was the major primary headache phenotype associated with palpation-induced pain in the masticatory muscles; d) migraine was mainly associated with bilateral AT palpation-induced pain; e) possible bruxism was mainly associated with bilateral masseter palpation-induced pain.

There are evidences supporting the association and the accepted comorbidity between primary headaches and TMD (11,16). Both disorders are clinically related, considering that the prevalence of TMD is higher in headache patients and vice-versa (17,18). Also, a recent study proposed multiple comorbidity among TMD, primary headaches and sleep bruxism (13). Despite this fact and the presumed pathophysiology for these relationships, i.e., common sensory innervation and central nervous system pathways via trigeminal nucleus caudalis, little is known regarding the role of palpation-induced muscle pain in the masticatory muscles on this multiple relationship. On the contrary, sound evidences indicate different patterns of pain distribution between TMD and TTH (19) and masticatory muscle pain sensitivity in TMD, migraine and TTH patients (6-8). Furthermore, this sensitivity is further increased when TMD is associated with headaches (20). Our results are in agreement with the above evidences and suggest different combinations of palpation-induced pain as being related to the diagnostic of painful TMD, primary headaches and bruxism, but also this multiple comorbidity is associated with a greater number of palpation-induced pain sites. 

The myogenous TMD is the most common type of painful TMD and it is a disabling disorder (14). In spite the fact of some circularity in the reasoning, since the pain to palpation is a criterion for pain-related TMD (1), it is important to note some differences in the pain distribution. The masseter muscle seems to be important for the diagnostic of myogenous TMD considering that the greatest odds ratio for myogenous TMD were related to the masseter palpation-induced pain. Our results are in line with evidences showing the masseter muscle as the most sensitive site in myofascial TMD pain patients (21). Furthermore, the pain drawings of myofascial TMD pain patients are similar to those of healthy participants under experimental masseter pain (19).

AT palpation-induced pain was associated with primary headache diagnosis. Likewise, despite some circular reasoning, we found different combinations of palpation-induced muscle pain between the primary headaches. Our results suggest that, even it is not completely understood, the nociceptive process in the masticatory muscles seems to play an important role in the TTH underlying mechanisms and the musculoskeletal physical exam could be considered relevant for the diagnostic (22). In fact, the palpation-induced pain is a prominent finding in the TTH population, especially chronic TTH (7). On the other hand, muscle factors are not considered essential in the well-established migraine pathophysiology; however, there are evidences of muscle pain as a presumed consequence of central sensitization process in migraineurs (8). Our results are similar with studies that pointed out masticatory muscle sensitivity in migraine patients, in particular, the bilateral AT sensitivity. We found bilateral AT palpation-induced pain associated with a 2.67 fold increase in odds of having migraine. Also, a possible puzzling aspect is the fact that the mechanical sensitivity reported by migraineurs can be result of the “natural” pericranial tenderness, typical of such population, regardless of the occurrence of migraine attacks. It has been demonstrated that PPT of women with migraine is reduced, even when no myogenous TMD is previously diagnosed (23). As stated before, this could be attributed to the well-known mechanisms of central and peripheral sensitization phenomena. Taken together, the differences in the distribution and patterns of palpation-induced muscle pain could be an important factor when assessing patients with TMD and primary headaches. The high degree of comorbidity among these disorders warrants further research about the role of muscle pain in this complex and often overlooked relationship. 

The significant, albeit weak, association between masseter palpation-induced pain and possible bruxism is an interest finding of this study. The majority of evidences on TMD-bruxism relationship support the association between bruxism and symptoms of muscle pain or fatigue (5). Indeed, the report of morning masticatory muscle pain/fatigue is considered a criterion for clinical diagnostic of sleep bruxism (24). Furthermore, epidemiological studies extensively present a positive association between bruxism and painful TMD, especially myogenous TMD (5). However, palpation-induced pain is not sine qua non for myofascial pain; so, it is not possible to presume from the above studies that bruxism is associated with pain to palpation. On the contrary, the association between palpation-induced pain and bruxism was not supported when polysomnographic recordings (PSG) were used as the diagnostic criteria, though one limitation of this study was the small sample size (25). Thus, we propose that our findings support the hypothesis of a possible muscle dysfunction in bruxism patients, which also present higher levels of pain-induced by function and higher muscle activity compared with asymptomatic controls (5). Nevertheless, the clinical value of pain to palpation on the diagnostic of bruxism remains to be established and sound conclusions are not possible considering that we used only the self-report for the bruxism assessment with no distinction between sleep and awake bruxism. 

This study’s strengths were the high degree of representativeness of the orofacial pain population in the clinical setting and the application of regression models for controlling the confounders and the overlap of diagnostics. It is also important to note that we adopted acceptable classification and assessment criteria for TMD, primary headaches and bruxism. On the other hand, the limitations of this study were mostly the lack of a rigorous and standardized protocol for evaluation, considering that there was no possibility of examiner’s blinding. Also, the participation of different examiners is also an important limitation. Finally, the cross-sectional characteristic does not allow cause-effect assumptions.

In conclusion, we endorse the importance of the routinely palpation-induced pain examination in the clinical assessment of pain disorders and emphasize its plausible value in terms of differential diagnosis among painful TMD, primary headaches and bruxism, nonetheless this aspect remains to be confirmed and further analyzed in future studies.
